# The impact of weather on COVID-19 pandemic

**DOI:** 10.1038/s41598-021-01189-3

**Published:** 2021-11-11

**Authors:** Michael Ganslmeier, Davide Furceri, Jonathan D. Ostry

**Affiliations:** 1 University of Oxford, 32 Wellington Square, Oxford, OX1 2ER UK; 2 International Monetary Fund, University of Palermo, RCEA, 1900 Pennsylvania Avenue NW, Washington, DC 20431 USA; 3 International Monetary Fund, CEPR, 1900 Pennsylvania Avenue NW, Washington, DC 20431 USA

**Keywords:** Environmental sciences, Environmental social sciences

## Abstract

Rising temperature levels during spring and summer are often argued to enable lifting of strict containment measures even in the absence of herd immunity. Despite broad scholarly interest in the relationship between weather and coronavirus spread, previous studies come to very mixed results. To contribute to this puzzle, the paper examines the impact of weather on the COVID-19 pandemic using a unique granular dataset of over 1.2 million daily observations covering over 3700 counties in nine countries for all seasons of 2020. Our results show that temperature and wind speed have a robust negative effect on virus spread after controlling for a range of potential confounding factors. These effects, however, are substantially larger during mealtimes, as well as in periods of high mobility and low containment, suggesting an important role for social behaviour.

## Introduction

The effect of weather on the spread of the coronavirus is one of the most investigated research questions since the onset of the pandemic^1–3^. Like other epidemic diseases, the trajectories in many countries show strong seasonal patterns with fewer cases during summer and more during winter. Although a range of studies has provided empirical evidence for the negative relationship between temperature and contagion^4–10^, several scholars come to contrasting conclusions by showing that the containment potential of weather differs substantially with respect to effect sizes, significance levels, weather indicators, regions, and time periods^11–15^.

Weather can influence virus contagion in two distinct ways. From an epidemiological standpoint, the survival and spread of a virus depends on the temperature of its environment. Since higher temperatures harm the lipid layer of the virus^10,16,17^, the viability of the SARS Coronavirus is substantially impaired at higher temperature levels^18^. From a behavioral perspective, weather alters mobility levels, social distancing, and location of social gatherings, which in turn affects the spread of the virus across individuals^19–21^. Thus, while the epidemiological channel implies lower cases during higher temperatures, the direction of the effect of weather through the social channel is not clear a priori, which may explain the conflicting results of previous empirical studies.

To investigate the weather-pandemic nexus, we collect a unique dataset covering 3376 counties in 114 states/regions from nine countries (Austria, Denmark, Finland, Ireland, Italy, Norway, Portugal, Sweden, and the United States) between 1st of January 2020 and 31st of December 2020, at a daily frequency. Using over 1.2 million observations and coverage of all seasons of the year, we examine the effect of weather on three alternative indicators^22,23^ which aim to capture the pandemic situation within a county on a given date: (1) (log) new cases; (2) number of new cases within the last 14 days per 100,000 habitants (notification rate); (3) (log) cases. As climatic indicators, we use hourly weather variables^24^ capturing: (1) temperature; (2) relative humidity; (3) wind speed; and (4) total precipitation in each county at a given date.

To quantify the effect of these weather variables, we use state-of-the-art econometric techniques that enable us to exploit comprehensive cross-county and within-county variation and achieve very high statistical precision in the empirical estimates. Such an exceptional regional granularity allows us to control for unobserved heterogeneity across counties—such as cultural factors—and regional-time-varying factors affecting the evolution of the pandemic—such as the imposition of lockdown measures, mask requirements and other factors affecting social distancing. In addition, we extend the literature by exploring alternative time lags between weather and virus cases—to take the delay between infection and reporting into account—and the effect of temperature at different hours of the day. This is essential since, as we show, weather affects contagion differently throughout the day depending on human activity (i.e. work, social gatherings).

## Results

### Temperature and wind speed have large containment effects

Our main estimation considers the effect of weather conditions measured at 12:00 local time 7 days prior to the case. Table 1 shows that temperature has a significantly negative effect on different case indicators. The effect is sizeable and highly significant [95% CI − 0.009 to − 0.003 for Table 1 column I]. This baseline estimate implies that an increase in temperature by 1 °C decreases (log) new cases by 0.006—that is, it reduces cases by 0.6%. This effect is sizable: an increase in the average temperature between February and March in our sample (about 5.5 °C) would lead to a decline of about 3.3% (5.5 × 0.6%) in the number of new cases per day. The specification captures the bulk of variation in observed new cases (R^2^ about 0.76)—suggesting that omitted variable bias is unlikely to be an important empirical issue. In addition, these results are robust across alternative epidemiological indicators used as dependent variables, which supports the validity of our findings (Table 1 columns II–III).

**Table 1:** The effect of different weather indicators at t-7 (at 12:00 local time) on different pandemic measures.

	(I)	(II)	(III)
	(log) new cases	New cases per 100 K capita	(log) cases
Temperature	− 0.0061*** (0.001)	− 4.0873*** (0.507)	− 0.0149*** (0.002)
Humidity	0.000015 (0.000)	− 0.6181*** (0.086)	− 0.0014*** (0.000)
Wind speed	− 0.0050*** (0.001)	− 1.6405*** (0.402)	− 0.0089*** (0.002)
Precipitation	− 0.4410 (0.270)	− 57.3877 (70.323)	− 0.7252** (0.366)
Constant	1.3282*** (0.034)	353.2458*** (13.111)	4.5083*** (0.064)
Observations	1,207,317	1,207,317	1,207,317
R-squared	0.76	0.70	0.94
County fixed effect	YES	YES	YES
State-date fixed effect	YES	YES	YES

The table reports the coefficients (with standard errors in parentheses) of four weather indicators based on the following regression model (baseline): ${y}_{i,s,t}={\beta }_{1}{Temperature}_{i,t-7}+{\beta }_{2}{Humidity}_{i,t-7}+{\beta }_{3}{WindSpeed}_{i,t-7}+{\beta }_{4}{Precipitation}_{i,t-7}+{\alpha }_{i}+{\gamma }_{s,t}+{\epsilon }_{i,s,t}$. The outcome variables are (log) new cases (I), number of new cases per 100,000 habitants within the last 14 days (II) and (log) cases (III). Standard deviations based on robust standard errors clustered at the county level in parentheses.

***,**,*Significance at 1, 5 and 10%, respectively; N = 1,207,317.

We find similar effects—in terms of size and significance—for wind speed where an increase from the first to the third quartile of its distribution lowers (log) new cases by 0.012. Although relative humidity is not significant in the baseline, we find statistically significant negative effects when using the notification rate and (log) cases as dependent variables. Precipitation is not significantly associated with the incidence in the baseline estimation, and thus, we are not able to confirm any containment potential emerging from rainfall. However, overall, these findings provide evidence that climatic factors play an important role for the pandemic trajectory: higher levels of temperature and wind bring both substantial containment effects.

**Figure 1:**
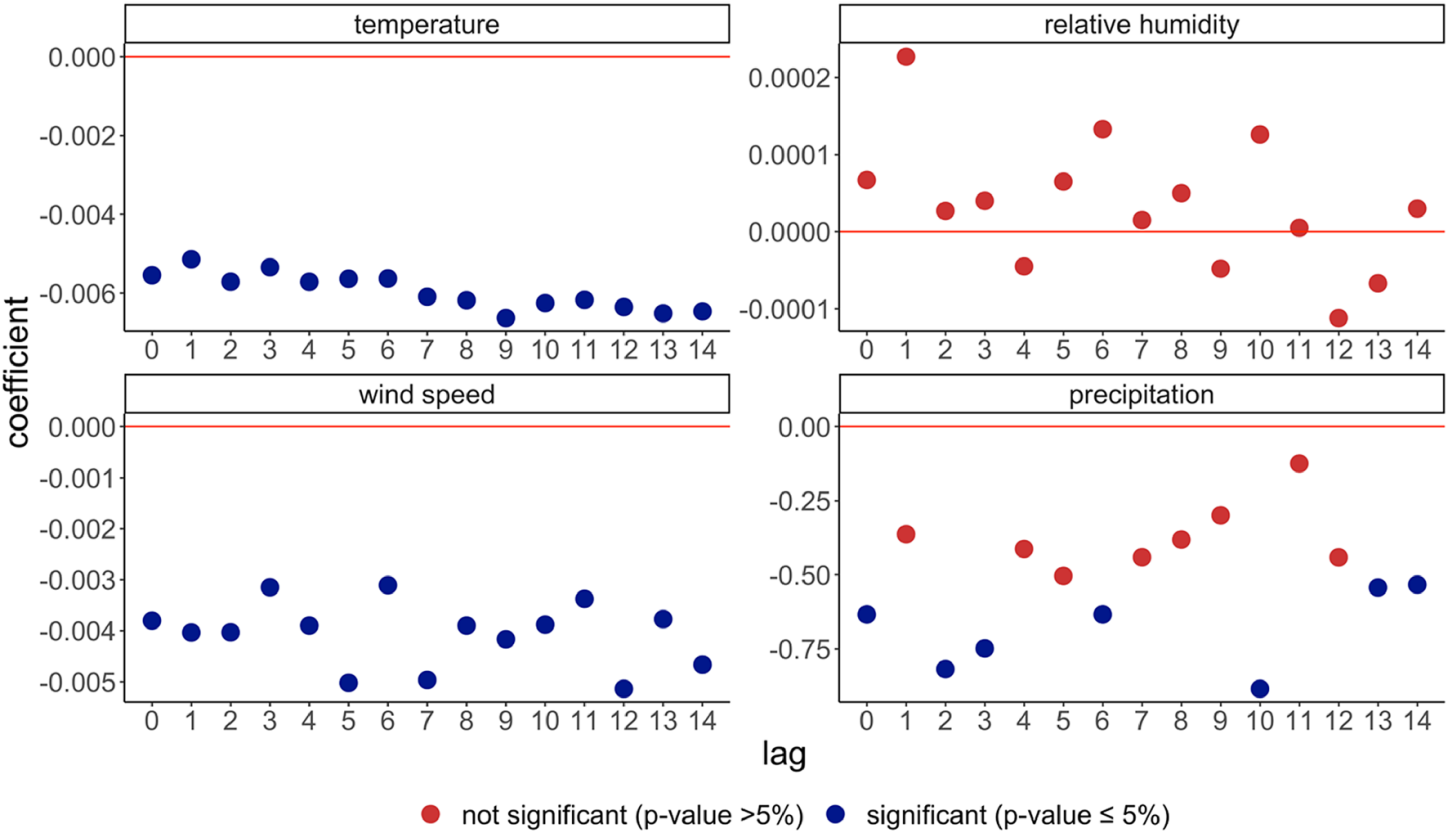
The effect of temperature and wind speed is significantly negative across different delay specifications. The outcome variable is (log) new cases. The x-axis shows different values of the delay time between 0 and 14 days. Standard deviations of the OLS estimations are based on robust standard errors clustered at the county level. Country and state-date fixed effects applied. N = 1,207,317.

Beyond these baseline results, we undertake two further extensions. First, in the baseline, we assumed that it takes, on average, 7 days between infection and reporting which is why we imposed a lag structure of 1 week on the weather variables. Although this assumption is comparable to other studies, we test whether the results differ when we change this delay structure. Here, we re-estimate the baseline for all delay assumptions between 0 and 14 days. As Figs. 1, S1 and S2 illustrates, the coefficients are similar in size and confidence intervals across lag structures (0–14) for temperature and wind speed.

Second, we account for potential non-linear weather effects due to epidemiological and social aspects of virus spread during spring and summer. To test this hypothesis, we re-estimate the baseline specification replacing the temperature term with a linear spline with a knot at 25 °C for the sample during the spring and summer months (1st of March until 31st of August 2020). In this way, we examine whether temperature increases below or above 25 °C carry larger containment potential. The results show that the containment effect of temperature below 25 °C is 15% larger than for temperature levels above 25 °C (Table 2) for incidence. This finding provides evidence that even small temperature increases should facilitate the fight against the virus during spring and summer months.

**Table 2:** The non-linear effect of different weather indicators at t-7 (at 12:00 local time) on different pandemic indicators.

	(I)	(II)	(III)
	(log) new cases	New cases per 100 K capita	(log) cases
Temperature spline < 25 °C	− 0.0116*** (0.002)	− 2.1171*** (0.395)	− 0.0183*** (0.003)
Temperature spline > 25 °C	− 0.0081*** (0.002)	− 0.0617 (0.505)	− 0.0089*** (0.003)
Humidity	− 0.0012*** (0.000)	− 0.1541*** (0.060)	− 0.0016*** (0.000)
Wind speed	− 0.0052*** (0.001)	− 0.1515 (0.336)	− 0.0074*** (0.002)
Precipitation	0.0244 (0.329)	− 3.2391 (71.118)	− 0.5390** (0.317)
Constant	1.2745*** (0.050)	147.0458*** (11.058)	4.2514*** (0.077)
Observations	595,251	595,251	595,251
R-squared	0.73	0.43	0.94
County fixed effect	YES	YES	YES
State-date fixed effect	YES	YES	YES

The outcome variables are (log) new cases (I), the number of new cases per 100,000 habitants within the last 14 days (II) and (log) cases (III). Standard deviations based on robust standard errors clustered at the county level in parentheses.

***,**,*Significance at 1, 5 and 10%, respectively; N = 595,251.

### The effects of weather are larger during working and mealtime hours

While these results are broadly in line with previous empirical studies, human activity changes throughout the day which is why the containment effect of weather may also differ across hours. To quantify this daytime-heterogeneity, we use the baseline specification but vary the time of the recording time of the weather variables between 00:00 and 23:00 local time. In this way, we have coefficients for each hour of the day which enables us to visualize the changing effect size and significance of the weather variables.

**Figure 2:**
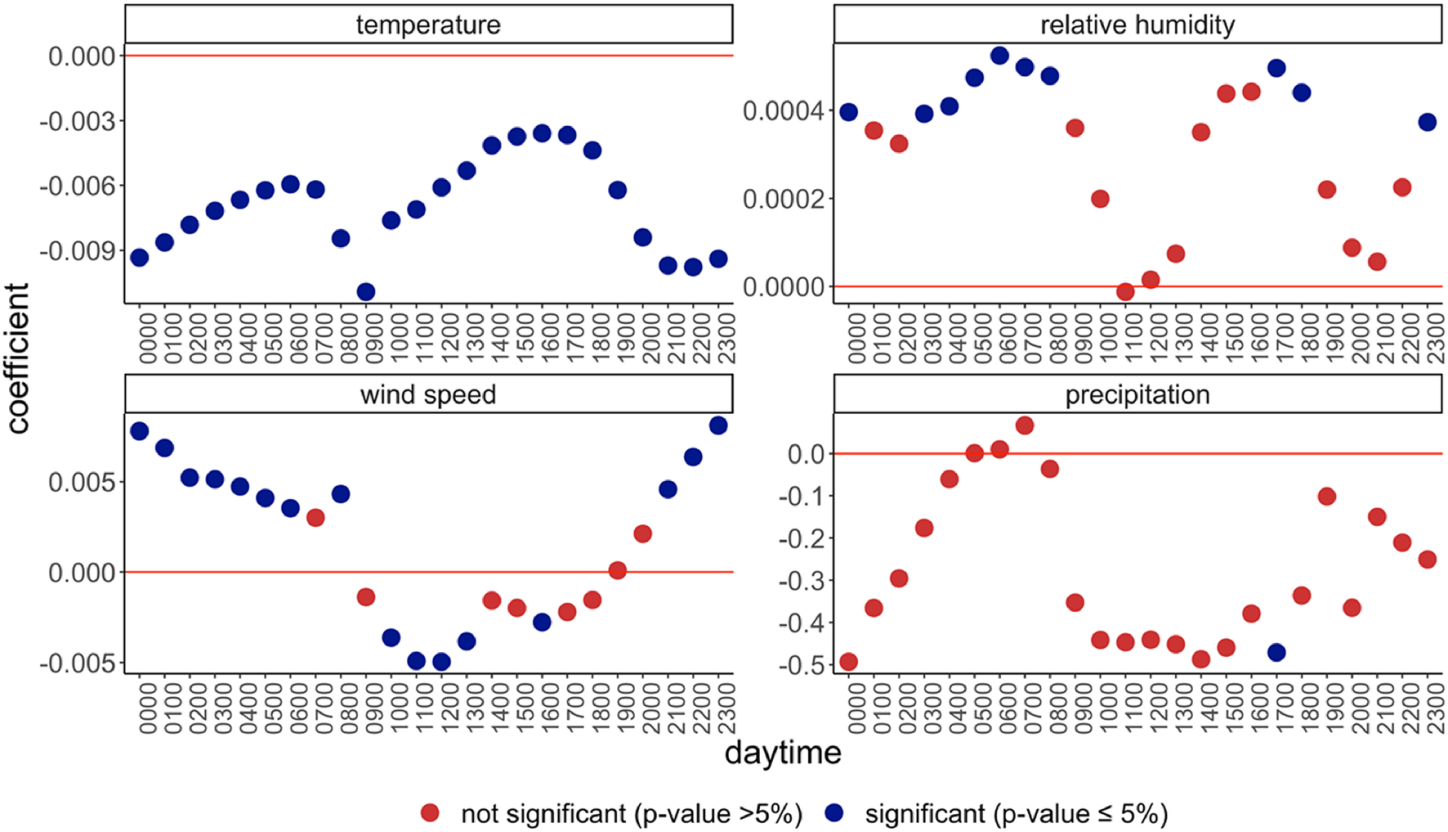
The effect of different weather indicators varies with daytime. The effect of different weather indicators at t-7 (at 12:00 local time) on new cases throughout the day. The outcome variable is (log) new cases. The x-axis shows the daytime between 00:00 and 23:00 (local time). Standard deviations of the OLS estimations are based on robust standard errors clustered at the county level. Country and state-date fixed effects applied. N = 1,207,317.

**Figure 3:**
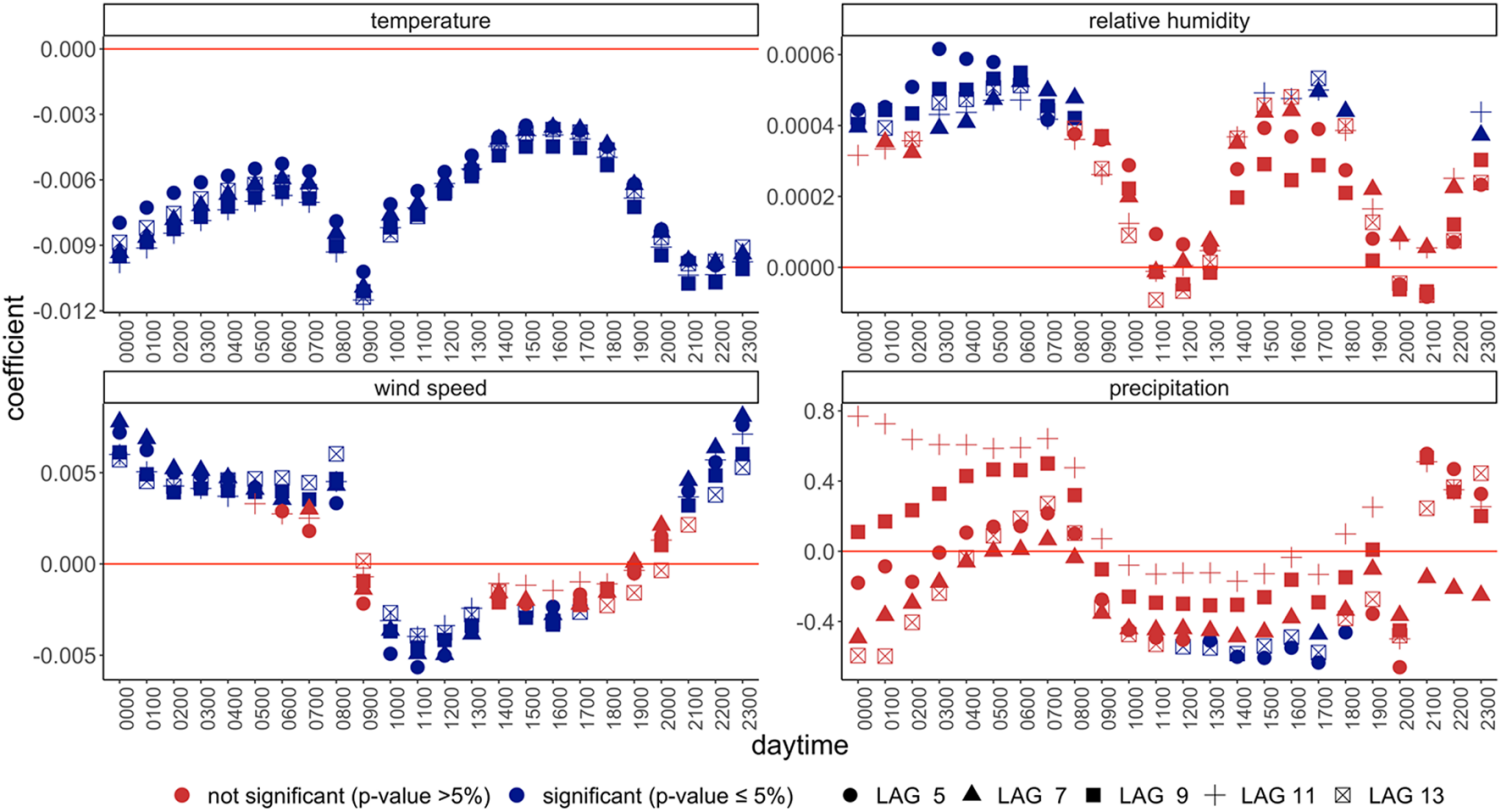
The effect of different weather indicators varies with daytime and is constant across different delay specifications. The effect of different weather indicators at t-7 (at 12:00 local time) on (log) new cases throughout the day with different delay assumptions. The outcome variable is (log) new cases. The x-axis shows different values of the daytime between 00:00 and 23:00 (local time). The shapes of the point estimates indicate the delay between weather and cases variables. Standard deviations of the OLS estimations are based on robust standard errors clustered at the county level. Country and state-date fixed effects applied. N = 1,213,842.

Our results show that the weather effects are larger during working hours and mealtimes (Fig. 2, see also Figs. S3 and S4). Specifically, our findings indicate that temperature has the largest negative impact on incidence at 9 a.m. and after 7 p.m., while wind has the largest negative impacts during working hours and lunch time (10 a.m.–1 p.m.). Interestingly, we find wave-shaped patterns for relative humidity indicating that high humidity in the early morning and in the early evening spurs contagion. We associate these changing patterns throughout the day with changes in the propensity to gather socially: while high temperature at dinner time enables outdoor meetings, high wind speed around lunchtime reduces mobility and social gatherings overall—both mechanisms carry containment potential and suppress the spread of the virus. Here, the baseline estimates include a single 7-day lag of the relevant weather indicator. However, these results are robust to other delay structures (Figs. 3, S5 and S6).

### The impact of weather is larger under high mobility levels and low containment measures

Previous research has indeed shown that both reducing physical mobility and containment measures have been key tools to limit the spread of the virus and reduce the number of fatalities around the world^19–21^. To test how these factors are mediated by the effects of weather, we include an interaction term consisting of temperature and one selected social distancing variable in the baseline specification.

Our results suggest that containment measures^25^ and high social distancing (lower mobility)^26^ have large effects in reducing the spread of the virus at lower temperatures, that is, when climatic conditions make the virus more susceptible to spread (Table 3). In other words, the effect of higher temperature on virus spread is larger during softer containment and higher mobility. The effects are largest for the stringency of containment policies [CI 95% 2.107–4.250] as well as workplace [95% CI − 2.577 to − 1.170], transit [95% CI − 3.235 to − 1.253] and retail-related mobility [95% CI − 2.675 to − 1.294]. We find similar effects when using (log) cases as the dependent variable (see Table S3).

**Table 3:** The effect of temperature at t-7 (at 12:00 local time) on different new cases mediated by containment policy and physical mobility.

	(I)	(II)	(III)	(IV)
	(log) new cases	(log) new cases	(log) new cases	(log) new cases
Temperature	− 0.0301*** (0.005)	− 0.0155*** (0.002)	− 0.0097*** (0.002)	− 0.0103*** (0.001)
Humidity	0.0001 (0.000)	− 0.0003 (0.000)	− 0.0003 (0.000)	− 0.0003 (0.000)
Wind speed	− 0.0033*** (0.001)	− 0.0053*** (0.001)	− 0.0058*** (0.001)	− 0.0056*** (0.001)
Precipitation	− 0.0907 (0.271)	− 0.1704 (0.258)	− 0.2379 (0.258)	− 0.2237 (0.258)
Temperature* interaction	3.1784*** (0.546)	− 1.8735*** (0.359)	− 2.2438*** (0.505)	− 1.9848*** (0.352)
Constant	− 0.0842 (0.243)	2.8603*** (0.261)	2.1635*** (0.149)	2.3432*** (0.150)
Interaction variable	Containment policy index	Mobility: workplace	Mobility: transit	Mobility: retail
Observations	1,207,317	1,043,565	1,038,491	1,043,275
R-squared	0.76	0.77	0.77	0.77
County fixed effect	YES	YES	YES	YES
State-date fixed effect	YES	YES	YES	YES

The outcome variable is (log) new cases. Column I includes the (standardized) interaction term between temperature and the containment health policy index provided by the Oxford Government Tracker; columns II–VI include (standardized) interaction terms between temperature and mobility indices provided Google. Standard deviations based on robust standard errors clustered at the county level in parentheses.

***,**,*Significance at 1, 5 and 10%, respectively; N = 1,207,317.

## Discussion

In summary, our results confirm the seasonality of coronavirus contagions by showing a negative association between temperature and the spread of the virus. In addition, the empirical evidence suggests that weather’s containment effects are largest at mealtimes, when weather has a substantial impact on the likelihood of social gatherings held indoors versus outdoors. Finally, our findings indicate that temperature have larger effects when containment measures are lifted, and mobility is higher.

There could be other factors influencing the impact of weather across countries and regions, such as cultural aspects, family and housing structure, and other variables influencing behaviors, including vaccination, etc. Incorporating further mediating factors may enable epidemiological studies to explain why warm weather in some regions does not have the same impact on incidence levels than in others. In addition, as many countries around the world have achieved high levels of vaccination, the importance of weather effects can change depending on the level of immunization in a specific geographical area. Thus, accounting for the share of vaccinated people within a country may be important to understand the changing effect of weather on COVID-19 cases.

Despite these limitations and possible extensions, our results have important policy implications for the future of containment policymaking as they suggest that rising temperature levels facilitates the fight against the virus. Thus, when approaching winter, more containment efforts are likely to be necessary, other things equal. To keep the restrictions as light as possible without losing control over the pandemic, implementing containment measures that limit certain kinds of social activities is likely to pay dividends in the fight against the virus without losing the will of individuals to comply with such measures.

## Methods

### Data

Our empirical estimates are based on a county-date panel dataset which covers 3376 counties in 114 states/NUTS2 regions in nine countries (Austria, Denmark, Finland, Ireland, Italy, Norway, Portugal, Sweden, and United States) over all seasons of 2020 on a daily basis. In total, the dataset has 1,235,616 observations, which is, to the best of our knowledge, the largest set of information ever used in the study of climatological effects on coronavirus spread. We have collected two kinds of variables, namely pandemic and weather variables. While the former one serves as our dependent variable, the latter ones enter our empirical analysis as explanatory factors.

Previous scholars who have investigated the effect of weather on virus spread have used different pandemic indicators which may explain the diverging results across studies. To test for heterogeneity across pandemic indicators, we use the most common measures used in the literature, namely (log) new cases, new cases per 100,000 capita in the previous 14 days and (log) cases. All of these variables are available with high regional granularity at the county level (for EU, NUTS3 regions) from the New York Times database for the US^22^ and ECDC for EU countries^23^.

For capturing the weather conditions in each county, we resort to data from the Copernicus database^24^. The EU-funded Copernicus satellite program compiles near-real-time data on climatological indicators since 1981 with a spatial resolution of ~ 28 km-by-28 km near the equator (0.25° × 0.25°). Based on reviewing previous literature about the weather-virus nexus, we focus on the four most used weather indicators in this strand of research, namely skin temperature, relative humidity, wind speed and total precipitation.

This high granularity with comprehensive time frequency enables us to merge very precise weather variables to the pandemic conditions in each location on given day. In addition, since the weather effect is not a purely epidemiological phenomenon but also a social one, one has to expect that the climatological impact on virus spread differs with changes in human and social behavior. This is true throughout the day as human activities and decision making depends strongly on the daytime and the weather conditions in this moment. Thus, to capture such changing patterns in human behavior through the day, hourly weather indicators are necessary. To the best of our knowledge, the present analysis is the first analysis of its kind that uses hourly measures.

Finally, to test the indirect of weather channeled through changes in human behavior, we use two kind of measures. On the one hand, containment policies have turned out to be successful instruments for governments to limit uncontrolled virus spread through social distancing rules. To capture how policy effectiveness depends on weather conditions, we use the national-level containment policy index constructed by the Oxford Government Response Tracker^25^. The database represents the largest collection of information on daily containment policies in over 180 countries since the onset of the pandemic. We use their overall containment and health index—which ranges from 0 (very loose containment policies) to 100 (very restrictive containment policies)—which consists of various sub-indices that capture lockdown restrictions, social distancing measures, contact tracing and testing policies amongst others. This measure enables us to assess the strictness of containment and health policies in a given country at a specific point in time.

On the other hand, mobility levels also played an important role in virus transmission. To understand this mechanism, Google made aggregated mobility indices available based on the movement of Android smartphones^26^. In detail, Google provides mobility indicators that range from − 100 to + 100 and which provide information how mobility to various avenues (e.g. groceries and pharmacy, retail and recreation, transit stations, parks, workplace, and residential) has changed compared to a weekday-specific baseline value that is based mobility levels during the period between January 3 and February, 2020^26^. We use these mobility indices that captures changes in mobility with respect to workplace, grocery/supermarket, transit, and retail. An overview on the variable definition, sources and key summary statistics can be found in Table S1.

### Empirical analysis

In the baseline estimate, we quantify the effect of the selected weather indicators on virus spread in the following form:

1 $${y}_{i,s,t}={\beta }_{1}{Temp}_{i,t-7}+{\beta }_{2}{Humidity}_{i,t-7}+{\beta }_{3}{WindSpeed}_{i,t-7}+{\beta }_{4}{Precip}_{i,t-7}+{\alpha }_{i}+{\gamma }_{s,t}+{\epsilon }_{i,s,t}$$

where ${y}_{i,s,t}$ is the pandemic indicator in county $i$ in region/state $s$ at date $t$. The dataset is a county-date panel where the climatological variables are recorded at 12:00 local time. To limit omitted variable bias, we include county ${(\alpha }_{i})$ and region-date fixed effects $({\gamma }_{s,t})$ to account for heterogeneity at the county-level (such as difference in cultural factors or the age structure of the population) and time-varying factors at the regional/state level (such as containment measures, mask orders, testing and contact tracing policies, etc.).

In contrast to most previous studies, the high regional granularity enables us to control for a multitude of factors affecting virus spread at the state level. Indeed, the very high R-Squared of our estimated models suggest that they capture the bulk of variations of cases at the county level. This gives us confidence that omitted variable bias is sufficiently addressed.

To test how the effect of social behavior is mediated through weather, we include an interaction term consisting of temperature and one selected social distancing variable in the baseline specification. The empirical model reads as follows:

2 $${y}_{i,s,t}={\beta }_{1}{({Mediator}_{c,t-7}*Temp}_{i,t-7})+\theta {X}_{i,t-7}+{\alpha }_{i}+{\gamma }_{s,t}+{\epsilon }_{i,s,t}$$

where ${y}_{i,s,t}$ is the pandemic indicator in county $i$ in region/state $s$ at date $t$; $X$ is a vector of the four weather indicators, namely temperature, relative humidity, wind speed and precipitation. Again, we include the same fixed effect structure for the reasons states above. The $Mediator$ terms include the national containment policy indicator from the Oxford Government Response Tracker for country $c$. For the mobility indices from Google, we are able to use state-level measures where $c=s$.

## Supplementary Information

Supplementary Information.

## Data Availability

All data and code used in the empirical analysis of the main text or the supplementary material are available upon request from the authors.
