# Supplementary material for: The impact of weather on COVID-19 pandemic
Source: Sci Rep. 2021 Nov 11;11:22027. doi: 10.1038/s41598-021-01189-3 (PMC8585954; doi:10.1038/s41598-021-01189-3)
Supplement: Supplementary file 1 — Supplementary Information. [file 41598_2021_1189_MOESM1_ESM.docx]

**The Impact of Weather on Covid-19 Pandemic**

Supplementary Information

**Michael Ganslmeier^1*^**, **Davide Furceri^2^, Jonathan D. Ostry^3^**

1 University of Oxford and University College London.
Address: University of Oxford, 32 Wellington Square, OX1 2ER Oxford, United Kingdom
Email: [michael.ganslmeier@spi.ox.ac.uk](mailto:michael.ganslmeier@spi.ox.ac.uk)

2International Monetary Fund. University of Palermo. RCEA.
Address: 1900 Pennsylvania Avenue NW, Washington, DC 20431, USA
Email: [dfurceri@imf.org](mailto:dfurceri@imf.org)

3International Monetary Fund. CEPR.
Address: 1900 Pennsylvania Avenue NW, Washington, DC 20431, USA
Email: [jostry@imf.org](mailto:jostry@imf.org)

**
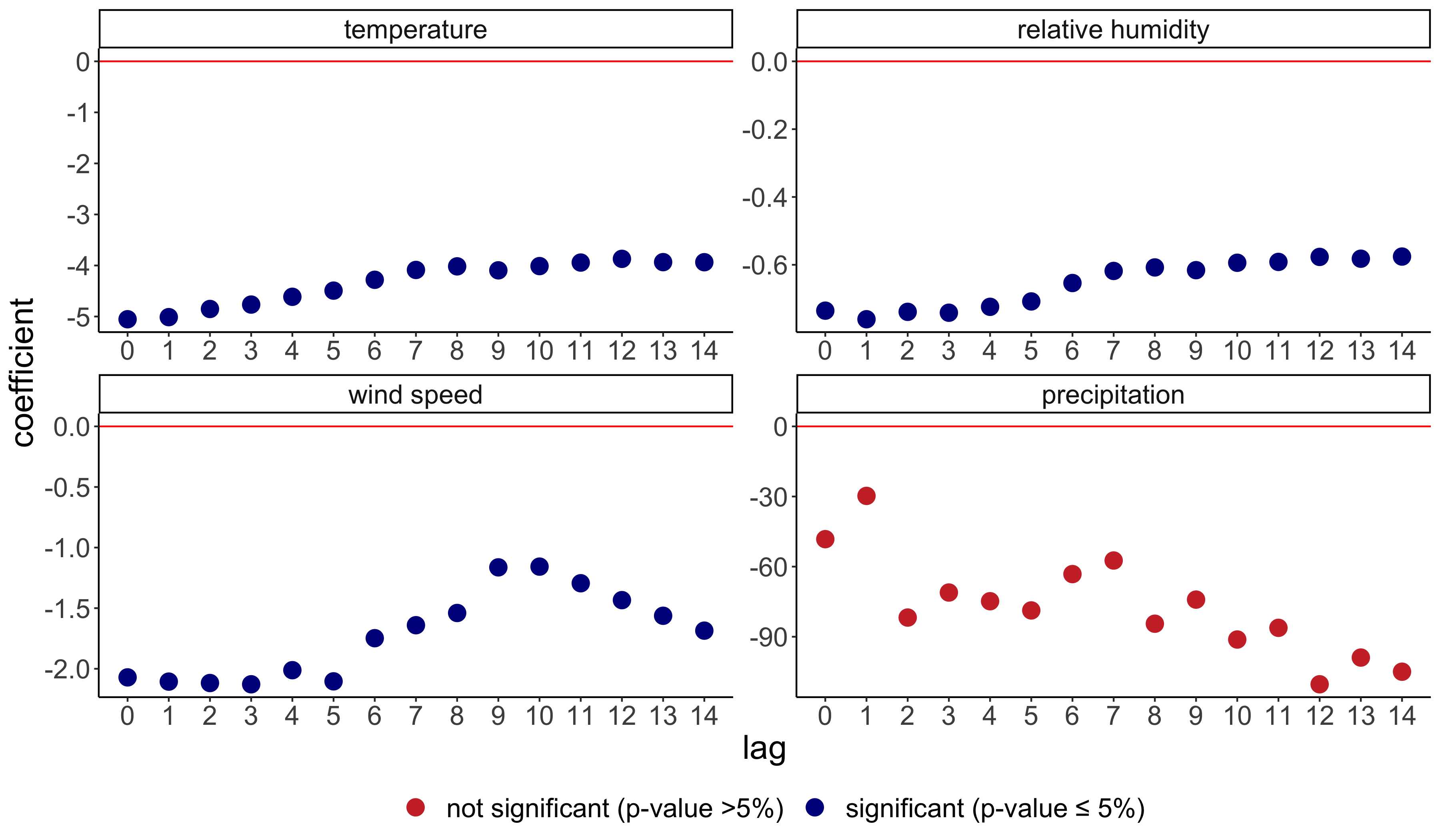
**

**Figure S1. The effect of different weather indicators on the case rate by different delay assumptions.** The outcome variable is the number of new cases per 100,000 habitants within the last 14 days. The x-axis shows different values of the delay time between 0 and 14 days. Standard deviations of the OLS estimations are based on robust standard errors clustered at the county level. Country and state-date fixed effects applied. N = 1,207,317

**
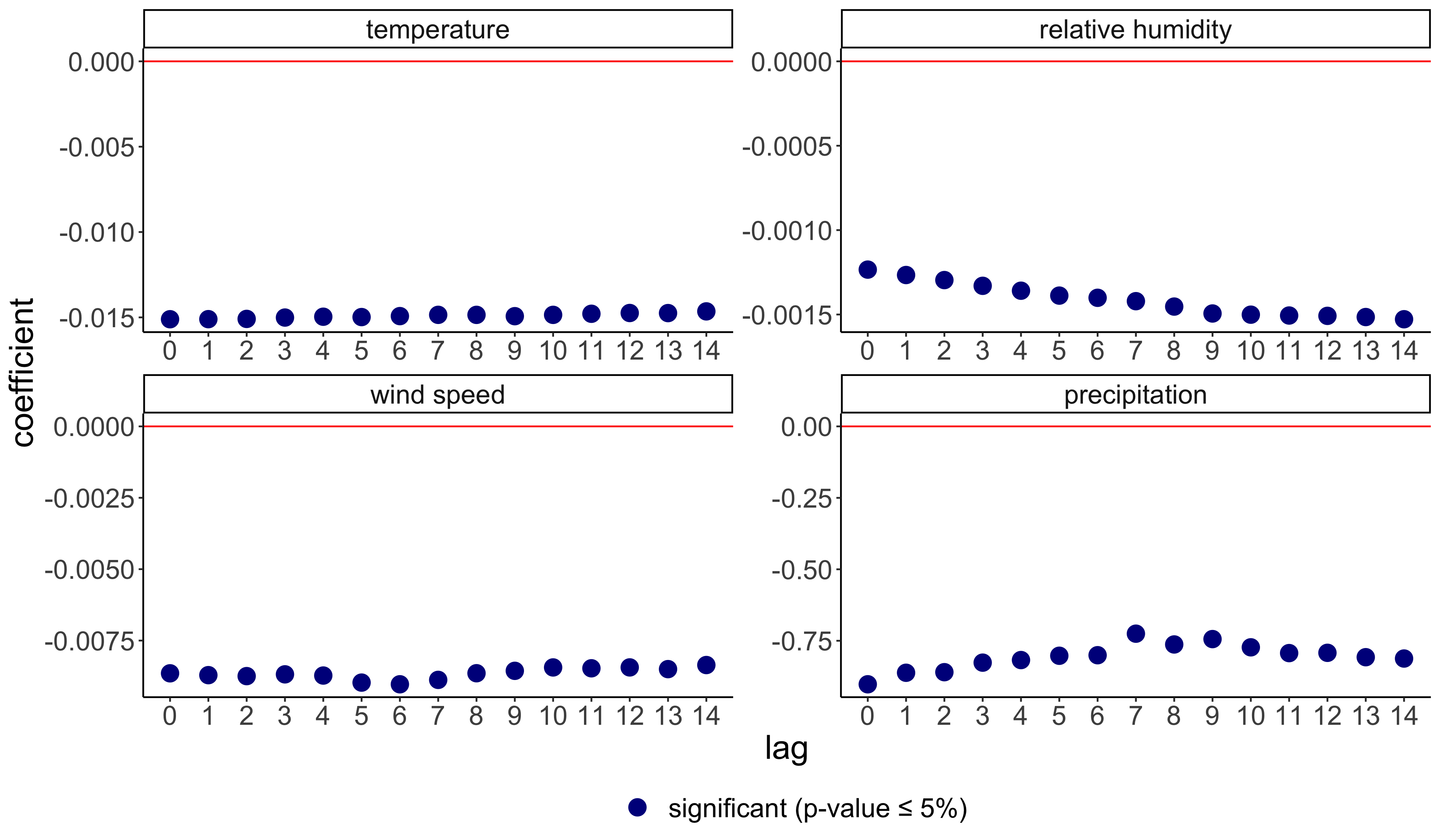
**

**Fig. S2. The effect of different weather indicators on cases by different delay assumptions.** The outcome variable is the number of cases. The x-axis shows different values of the delay time between 0 and 14 days. Standard deviations of the OLS estimations are based on robust standard errors clustered at the county level. Country and state-date fixed effects applied. N = 1,207,317


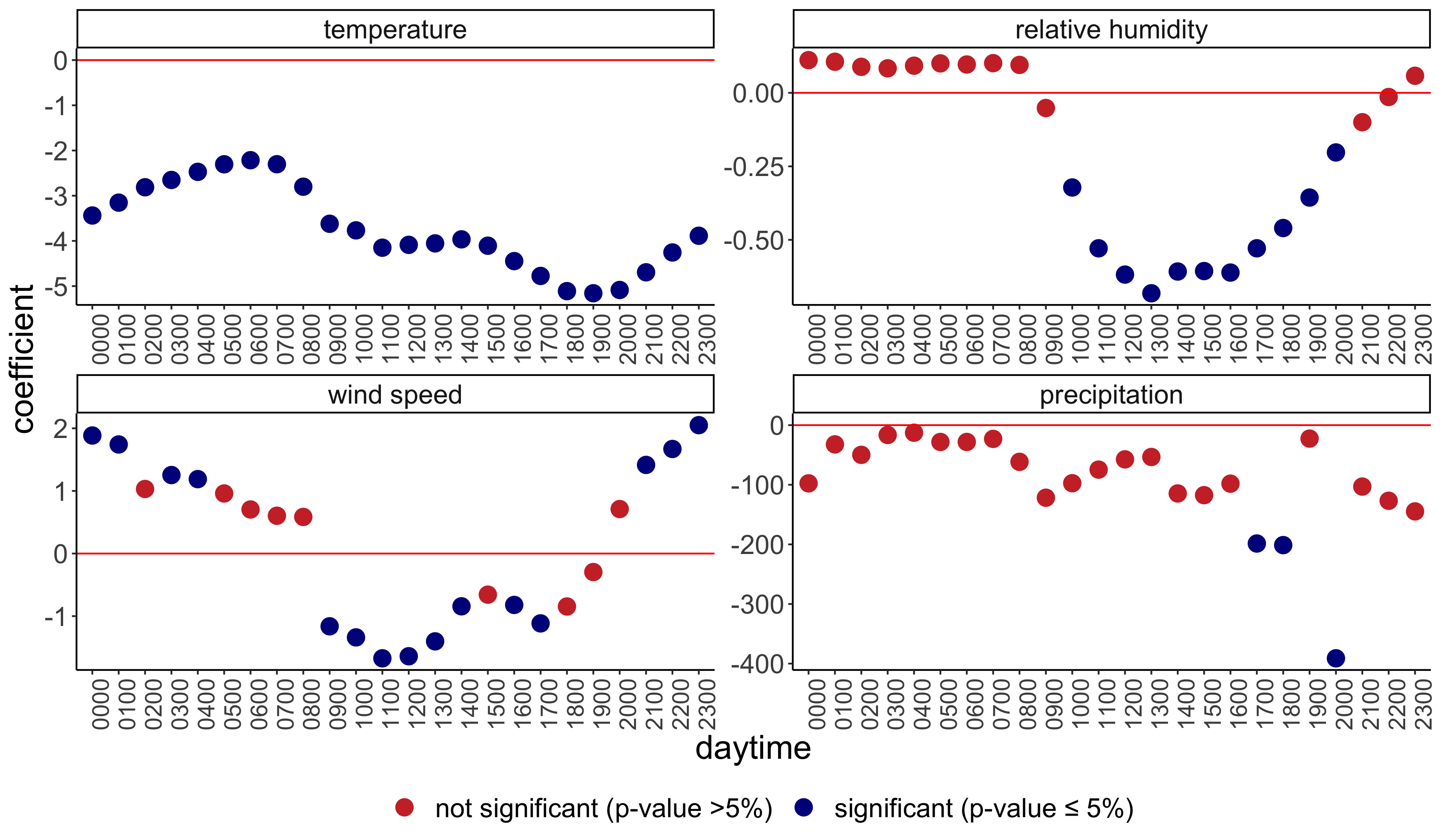


**Fig. S3. The effect of different weather indicators at t-7 (at 12:00 local time) on the cases rate throughout the day.** The outcome variable is the number of new cases per 100,000 habitants within the last 14 days. The x-axis shows different values of the daytime between 00:00 and 23:00 (local time). Standard deviations of the OLS estimations are based on robust standard errors clustered at the county level. Country and state-date fixed effects applied. N = 1,207,317


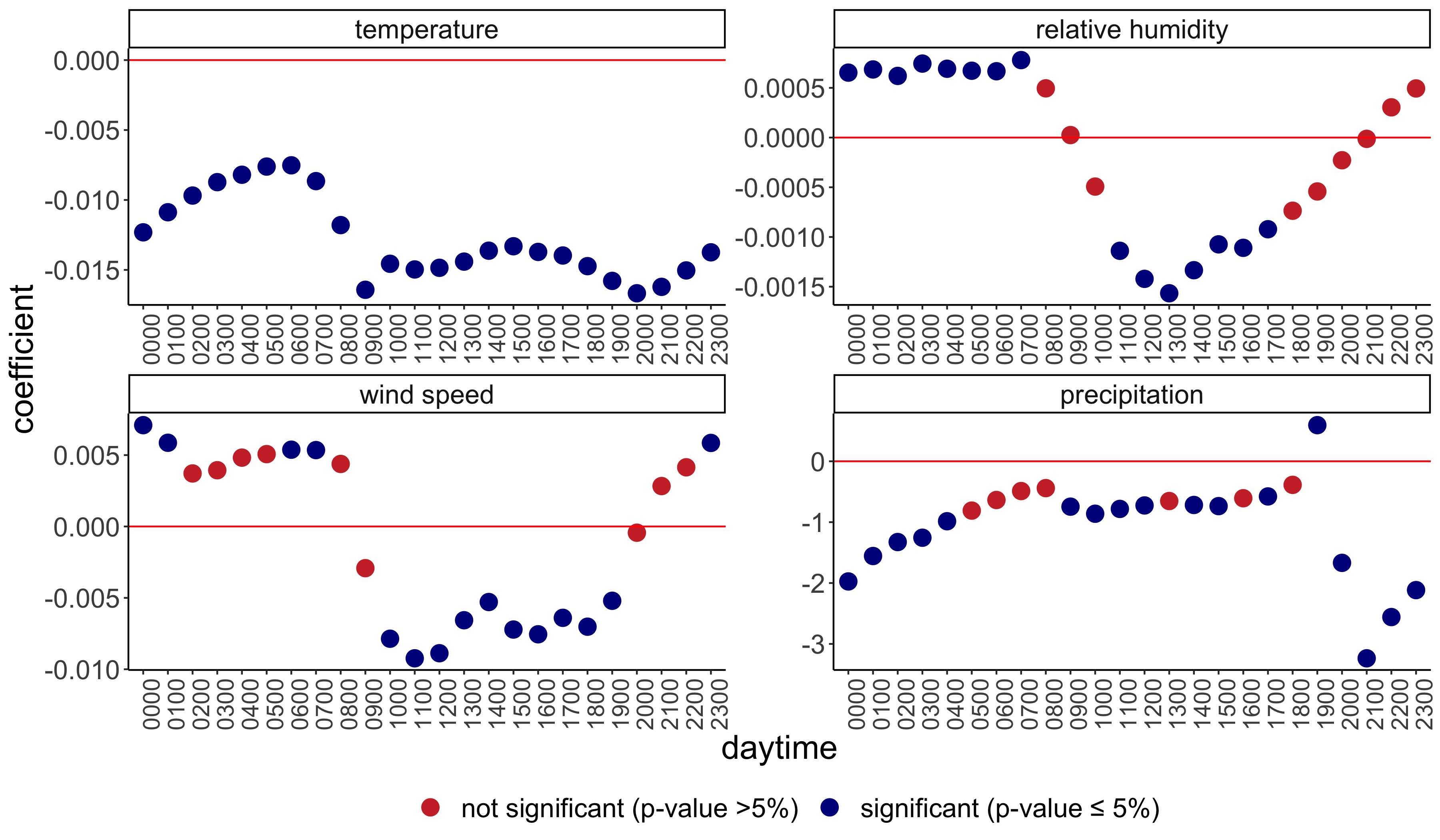


**Fig. S4. The effect of different weather indicators at t-7 (at 12:00 local time) on cases throughout the day.** The outcome variable is the number of cases. The x-axis shows different values of the daytime between 00:00 and 23:00 (local time). Standard deviations of the OLS estimations are based on robust standard errors clustered at the county level. Country and state-date fixed effects applied. N = 1,207,317


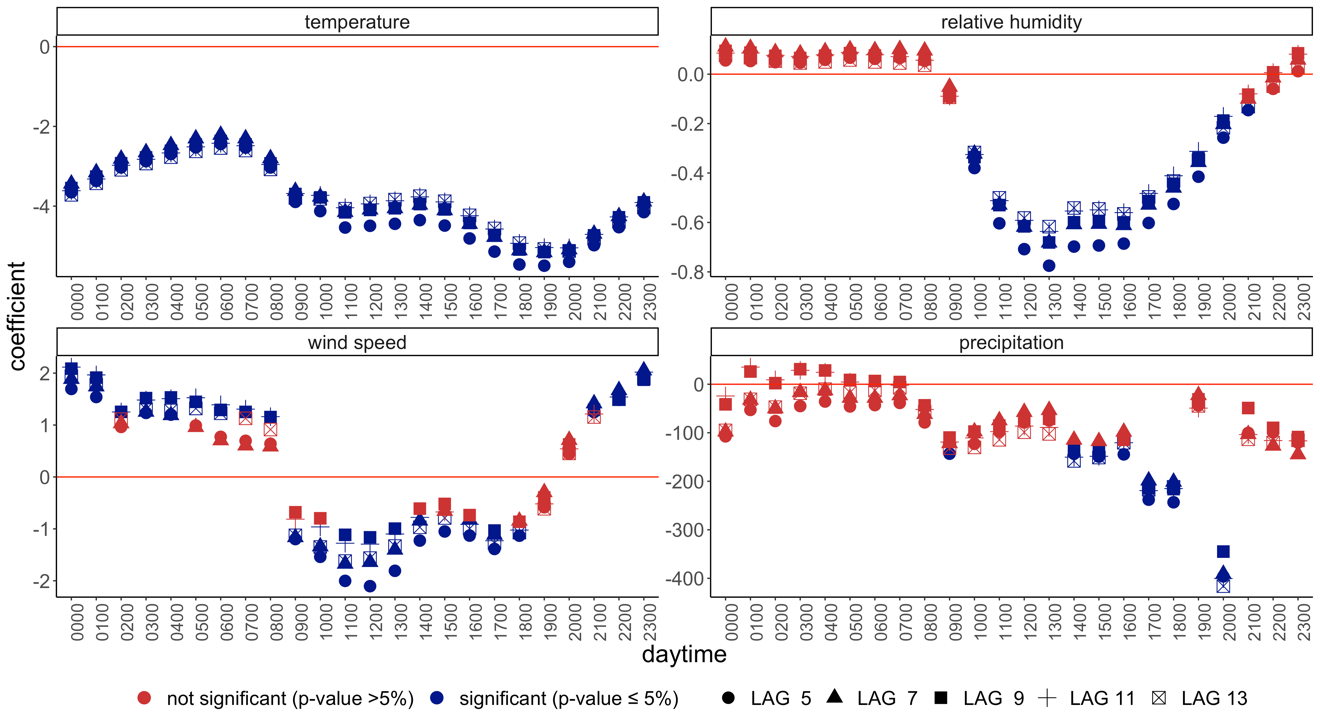


**Fig. S5. The effect of different weather indicators at t-7 (at 12:00 local time) on the cases rate throughout the day with different delay assumptions.** The outcome variable is the number of cases. The x-axis shows different values of the daytime between 00:00 and 23:00 (local time). The shapes of the point estimates indicate the delay between weather and cases variables. Standard deviations of the OLS estimations are based on robust standard errors clustered at the county level. Country and state-date fixed effects applied. N = 1,213,842


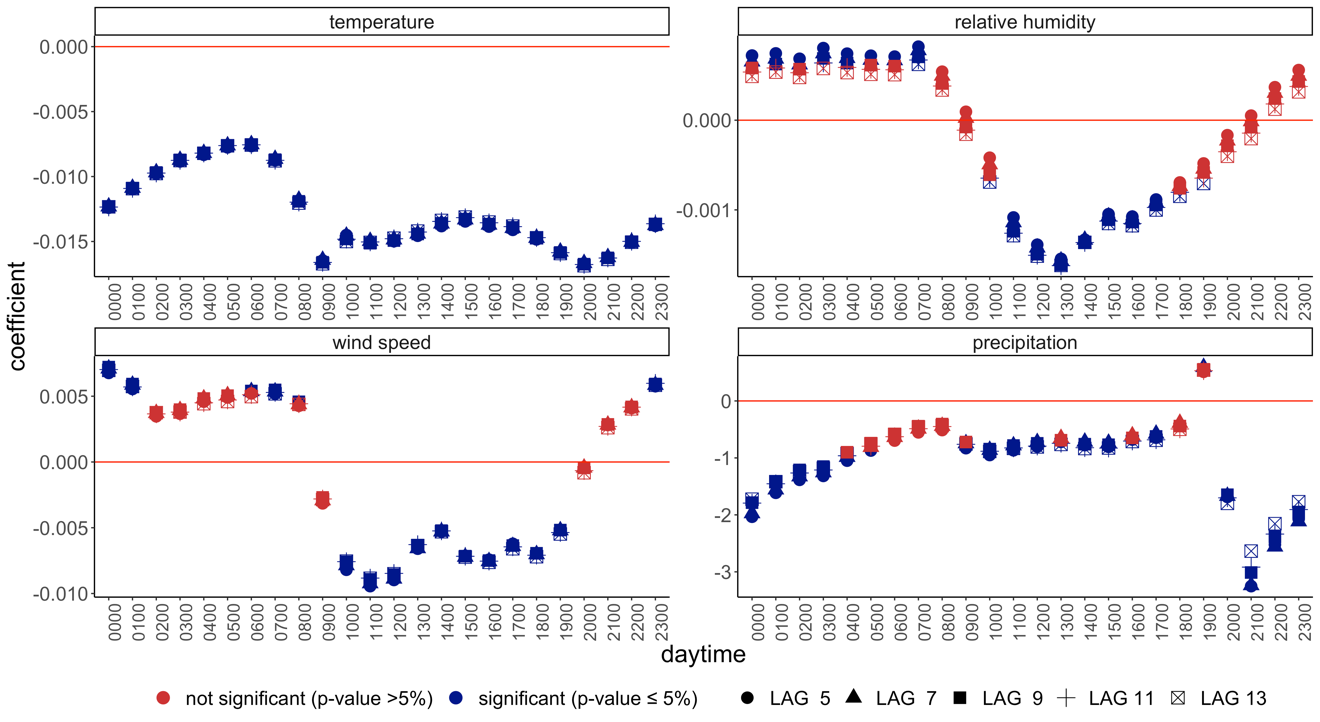


**Fig. S6. The effect of different weather indicators at t-7 (at 12:00 local time) on cases throughout the day with different delay assumptions.** The outcome variable is the number of cases. The x-axis shows different values of the daytime between 00:00 and 23:00 (local time). The shapes of the point estimates indicate the delay between weather and cases variables. Standard deviations of the OLS estimations are based on robust standard errors clustered at the county level. Country and state-date fixed effects applied. N = 1,213,842

**Table S1. Definitions, sources, and summary statistics**

| **Definition** | **Source** | **N  (Tsd.)** | **Mean** | **SD** | **Min** | **Max** |
| --- | --- | --- | --- | --- | --- | --- |
| (log) new cases | NY Times (US) ECDC (EU) | 1232 | 1.185 | 1.532 | 0 | 10.28 |
| new cases/100K capita  within last 14 days | NY Times (US) ECDC (EU) | 1236 | 232.1 | 423.9 | 0 | 13.265 |
| (log) cases | NY Times (US) ECDC (EU) | 1236 | 4.052 | 3.066 | 0 | 13.56 |
| precipitation (at 12:00) | Copernicus | 1235 | 0.0019 | 0.0054 | 0 | 0.224 |
| skin temperature (at 12:00) | Copernicus | 1235 | 18.15 | 11.49 | -45.78 | 60.11 |
| wind speed (at 12:00) | Copernicus | 1235 | 3.161 | 1.911 | 0.0025 | 20.43 |
| relative humidity (at 12:00) | Copernicus | 1235 | 57.76 | 19.37 | 3.530 | 100.0 |
| containment health index | Oxford Policy Tracker | 1236 | 53.25 | 23.39 | 0 | 84.29 |
| mobility index: grocery | Google | 1070 | -1.248 | 12.29 | -96 | 217 |
| mobility index: retail | Google | 1070 | -14.91 | 16.82 | -97 | 105 |
| mobility index: transit | Google | 1065 | -18.89 | 20.50 | -93 | 199 |
| mobility index: workplace | Google | 1070 | -27.10 | 15.76 | -92 | 40 |

**Table S2. The effect of temperature at t-7 (at 12:00 local time) on the case rate mediated by containment policies and mobility levels**

|  | (I) | (II) | (III) | (IV) |
| --- | --- | --- | --- | --- |
|  | new cases  per 100K capita | new cases per 100K capita | new cases per 100K capita | new cases per 100K capita |
|  |  |  |  |  |
| temperature | -3.2087*** | -6.9401*** | -5.8386*** | -6.6165*** |
|  | (0.767) | (0.688) | (0.721) | (0.733) |
| humidity | -0.6216*** | -1.0060*** | -1.0134*** | -1.0055*** |
|  | (0.085) | (0.118) | (0.118) | (0.118) |
| wind speed | -1.7007*** | -2.6350*** | -2.5556*** | -2.6342*** |
|  | (0.412) | (0.486) | (0.483) | (0.483) |
| precipitation | -70.2141 | -177.4793** | -188.4149** | -180.7579** |
|  | (72.967) | (84.709) | (84.161) | (84.415) |
| temperature* interaction | -116.3707 | -60.7055 | 430.2065** | 2.7674 |
|  | (81.281) | (49.001) | (168.971) | (91.452) |
| Constant | 404.9576*** | 515.0313*** | 349.5402*** | 470.5581*** |
|  | (38.111) | (38.238) | (52.043) | (45.718) |
|  |  |  |  |  |
| Interaction variable | containment policy index | mobility: workplace | mobility:  transit | mobility:  retail |
| Observations | 1,207,317 | 1,043,565 | 1,038,491 | 1,043,275 |
| R-squared | 0.70 | 0.69 | 0.69 | 0.69 |
| County Fixed-Effect | YES | YES | YES | YES |
| State-Date Fixed-Effect | YES | YES | YES | YES |

Note: The outcome variable is new cases per 100,000 habitants within the previous 14 days. We use a single 7-day lag variable for each weather indicator and the interaction term as independent variables. Column I includes the interaction term between temperature and the containment health policy index provided by the Oxford Government Tracker; column II-VI includes interaction terms between temperature and mobility indices provided Google. Standard deviations based on robust standard errors clustered at the county level in parentheses. ***,**,* denote significance at 1, 5 and 10 percent, respectively; N = 1,207,317

**Table S3. The effect of temperature at t-7 (at 12:00 local time) on cases mediated by containment policies and mobility levels**

|  | (I) | (II) | (III) | (V) |
| --- | --- | --- | --- | --- |
|  | (log)  cases | (log)  cases | (log)  cases | (log)  cases |
|  |  |  |  |  |
| temperature | -0.0500*** | -0.0270*** | -0.0194*** | -0.0183*** |
|  | (0.007) | (0.003) | (0.002) | (0.002) |
| humidity | -0.0013*** | -0.0015*** | -0.0014*** | -0.0014*** |
|  | (0.000) | (0.000) | (0.000) | (0.000) |
| wind speed | -0.0065*** | -0.0083*** | -0.0090*** | -0.0087*** |
|  | (0.002) | (0.002) | (0.002) | (0.002) |
| precipitation | -0.2123 | -0.1918 | -0.2910 | -0.2824 |
|  | (0.362) | (0.233) | (0.236) | (0.234) |
| temperature* interaction | 4.6541*** | -2.8674*** | -4.0109*** | -2.7016*** |
|  | (0.879) | (0.509) | (0.809) | (0.554) |
| Constant | 2.4402*** | 7.1122*** | 6.2067*** | 6.1808*** |
|  | (0.400) | (0.363) | (0.237) | (0.234) |
|  |  |  |  |  |
| Interaction variable | containment policy index | mobility: workplace | mobility:  transit | mobility:  retail |
| Observations | 1,207,317 | 1,043,565 | 1,038,491 | 1,043,275 |
| R-squared | 0.94 | 0.95 | 0.95 | 0.95 |
| County Fixed-Effect | YES | YES | YES | YES |
| State-Date Fixed-Effect | YES | YES | YES | YES |

Note: The outcome variable is (log) cases. We use a single 7-day lag variable for each weather indicator and the interaction term as independent variables. Column I includes the interaction term between temperature and the containment health policy index provided by the Oxford Government Tracker; column II-VI includes interaction terms between temperature and mobility indices provided Google. Standard deviations based on robust standard errors clustered at the county level in parentheses. ***,**,* denote significance at 1, 5 and 10 percent, respectively; N = 1,207,317
